# Clinical relevance of serum-derived exosomal messenger RNA sequencing in patients with non-Hodgkin lymphoma

**DOI:** 10.7150/jca.69639

**Published:** 2022-02-21

**Authors:** Yeong Hak Bang, Joon Ho Shim, Kyung Ju Ryu, Yeon Jeong Kim, Myung Eun Choi, Sang Eun Yoon, Junhun Cho, Bon Park, Woong-Yang Park, Won Seog Kim, Seok Jin Kim

**Affiliations:** 1 Department of Digital Health, Samsung Advanced Institute for Health Sciences and Technology, Sungkyunkwan University, Seoul, Korea.; 2 Department of Health Sciences and Technology, Samsung Advanced Institute for Health Sciences and Technology, Sungkyunkwan University, Seoul, Korea.; 3 Samsung Genome Institute, Samsung Medical Center, Seoul, Republic of Korea.; 4 Division of Hematology and Oncology, Department of Medicine, Samsung Medical Center, Sungkyunkwan University School of Medicine, Seoul, Korea.; 5 Department of Pathology and Translational Genomics, Samsung Medical Center, Sungkyunkwan University College of Medicine, Seoul, Korea.

**Keywords:** Liquid biopsy, exosome, messenger RNA, non-Hodgkin lymphoma

## Abstract

**Background:** The clinical utility of mRNA cargo in exosomes is unclear, although exosomes have potential as non-invasive biomarkers. This study aimed to investigate the feasibility of exosomal mRNA sequencing for monitoring disease status and predicting outcomes in non-Hodgkin lymphoma (NHL) patients.

**Methods:** Exosomes were isolated from archived serum samples of 33 patients with NHL who were registered into our prospective cohort: diffuse large B-cell lymphoma (DLBCL, n = 17), intravascular B-cell lymphoma (IVL, n = 1), primary mediastinal large B-cell lymphoma (PMBL, n = 4), follicular lymphoma (FL, n = 3), mantle cell lymphoma (MCL, n = 3), and extranodal NK/T-cell lymphoma (ENKTL, n = 5). Exosomal mRNA sequencing was performed, and its concordance with clinical course was analyzed and compared with those of circulating tumor DNA (ctDNA) mutations.

**Results:** Exosomal mRNA sequencing was performed successfully in 26 cases (79%, 26/33), whereas the remaining seven cases were not completed due to their small amount of RNA. The exosomal mRNA sequencing of DLBCL showed gene expression profiles consistent with activated B-cell-like and germinal center type. The longitudinal assessment of exosomal mRNA sequencing results in accordance with the clinical course showed that the post-treatment changes of exosomal mRNA expression were more consistent with treatment outcome than were those of ctDNA mutations. In particular, the exosomal mRNA expression of genes such as *BCL2* and *BCL6* was increased at the time of disease progression in DLBCL and FL patients.

**Conclusions:** This study demonstrated the feasibility of exosomal mRNA expression profiles as a biomarker for NHL patients. Our results might provide the rationale for studies to explore the potential of exosomal mRNA as a biomarker in NHL patients.

## Introduction

Non-Hodgkin lymphoma (NHL) is the most common form of blood cancer and is a curable disorder by chemotherapy. However, a substantial number of NHL patients relapse even after radiological imaging resolution of tumor lesions at the end of treatment. This could imply the existence of residual lymphoma cells that survived after chemotherapy, and the occurrence of treatment resistance during chemotherapy could explain the persistence of viable lymphoma cells after treatment. Thus, detection of minimal residual disease (MRD) during or after chemotherapy could guide additional treatments for patients at high risk of relapse 1, 2. Liquid biopsy using peripheral blood is a convenient repeatable way to assess MRD in lymphoma patients through detection of lymphoma-derived materials in blood 3, 4. Indeed, plasma-circulating tumor DNA (ctDNA) has been developed to serve as a diagnostic tool of MRD detection in NHL patients because it can represent indirectly the presence of primary tumor 5-7. However, ctDNA might not reflect disease status because a proportion derives from apoptotic dying cells rather than from viable tumor cells 8. Furthermore, ctDNA analysis could provide information confined to mutation profiles rather than profiles of gene expression.

Extracellular vesicles (EVs) are abundant in almost all human biological fluids, including blood. Thus, EVs have been thought to hold tremendous potential as non-invasive biomarkers because the information within them could reflect their originating cells 9, 10. Diverse types of EVs could exist because size, origin, and biogenesis can be variable. Among them, exosomes, also known as 'small extracellular vesicles,' are the smallest, having a dimeter of 30-150 nm 11. After intraluminal vesicles are formed primarily within multivesicular bodies, fusion of multivesicular bodies with the plasma membrane could lead to release of exosomes into the extracellular space 12. Tumor cells could release exosomes constitutively into blood, and tumor-derived exosomal cargo could contain proteins, DNA, messenger RNA (mRNA), and microRNA related with parental tumor cells 13, 14. Nevertheless, most studies have focused on exosomal microRNA because exosomes contain mainly small RNA such as microRNA 15, 16. However, exosomal mRNA also could be an important biomarker because its presence inside exosomes was reported to represent parental tumor cells, although exosomes contain a small amount of mRNA 17. Recently, the clinical relevance of exosomal mRNA expression was suggested in patients with metastatic prostate cancer 18. Given the importance of gene expression in the pathogenesis and classification of lymphomas, analysis of mRNA inside exosomes actively released from lymphoma cells might be useful for detection of MRD. However, the feasibility of exosomal mRNA as a biomarker has never been studied in NHL patients. Thus, we performed mRNA sequencing with serum-derived exosomes obtained from NHL patients to explore the clinical relevance of exosomal mRNA as a biomarker for monitoring disease status.

## Methods

### Patients

**Figure 1:**
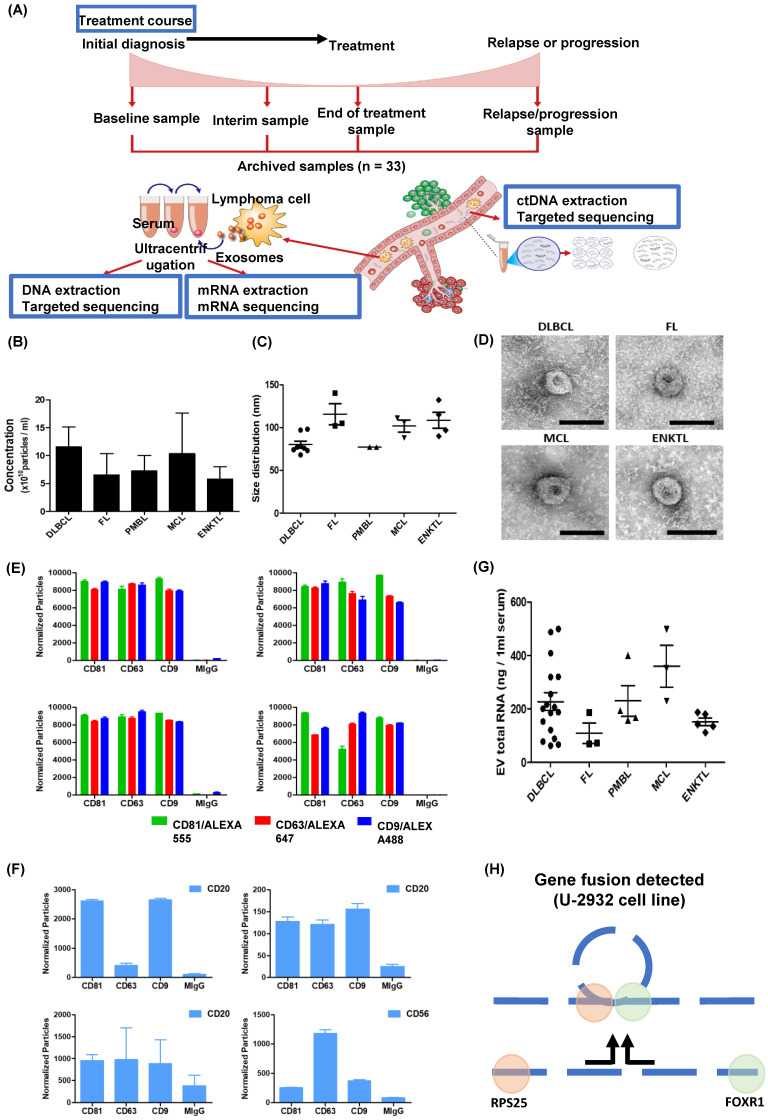
** Analysis of exosomal mRNA expression in patients with non-Hodgkin lymphoma. (A)** The scheme illustrating the process of the study method and cohort: Exosomal mRNA and matched ctDNA are extracted from blood. Serial changes were analyzed according to the treatment course. **(B, C)** Concentration and size distribution profiles of exosomes measured by nanoparticle tracking analysis. **(D)** Transmission electron microcopy image of exosomes from DLBCL, FL, MCL, and ENKTL patients. Scale bar, 100 nm. **(E)** Number of particles of CD81, CD63, and CD9-positive exosomes in non-Hodgkin lymphoma. Three-color fluorescence detection of exosomal markers (CD81, CD63, CD9) using secondary anti-body staining with a cocktail of anti-CD81, anti-CD63, and anti-CD9 labeled with Alex 488, 555, and 647, respectively, using ExoView. **(F)** Number of particles of CD20 in patients with B-cell lymphoma or CD56-positive exosomes in patients with NK/T-cell lymphoma. Exosomes captured on the chip by anti-CD81, anti-CD63, or anti-CD9 were detected by anti-CD20 or anti-CD56 and labeled with APC using ExoView. **(G)** The concentrations of total RNA in serum exosomes from DLBCL, FL, PMBL, MCL, and ENKTL patients. **(H)** Fusion transcripts of 5′ Ribosomal Protein S25 (RPS25) with FOXR1 were detected in the EV-derived mRNA from DLBCL cell line U-2932.

This study aimed to explore the feasibility of serum-derived exosomal mRNA expression profiles as biomarkers for NHL patients. Thus, we isolated exosomes from archived serum samples of NHL patients and performed mRNA sequencing using RNA extracted from exosomal cargo. Eligible patients were enrolled in our single-center, prospective cohort study (NCT03117036) that was approved by the institutional review board (No. 2016-11-040). After obtaining written informed consent, we collected serum samples at enrollment of the prospective cohort (baseline), during interim evaluations (interim), at the final response evaluation (end of treatment), and/or at the time of relapse or progression (progression). Enrolled patients were treated and monitored in a real-life context, and collected serum samples were stored at -80 °C until analysis. The survival status of all enrolled patients was updated regularly, and the last update regarding survival and disease status was completed in August 2021. Of the patients enrolled in the aforementioned prospective cohort study, the samples of 33 were analyzed in this study according to the following inclusion criteria: (1) treated with curative intent; (2) data available for treatment response and survival outcome; (3) archived serum samples that were obtained at baseline, interim, end of treatment, and/or relapse/progression; and (4) data for ctDNA mutation profiles (Figure 1A). In this study, the feasibility of exosomal mRNA sequencing was evaluated in each subtype, and the longitudinal changes in exosomal mRNA expression were compared with the clinical course as well as ctDNA mutations using data from our previous study 19.

### Isolation of exosomes by ultracentrifugation

The isolation of exosomes from serum was performed according to the sequential centrifugation protocol. Thus, serum was centrifuged differentially at 2000xg at 4 °C for 10 min and 10,000xg at 4 °C for 30 min. Serum was diluted in 3 ml of phosphate-buffered saline (PBS) and filtered through a 0.2-μm pore filter. Afterward, ultracentrifugation at 110,000xg at 4 °C was performed for 120 min. Next, the exosome pellet was suspended in 11 ml of PBS and ultracentrifuged at 110,000xg at 4 °C for 70 min. The final exosome pellet was suspended in 200 μL of PBS.

### Transmission electron microscopy (TEM) and nanoparticle tracking analysis (NTA)

Isolated exosomes were fixed in 4% paraformaldehyde and transferred onto Formvar-carbon-coated EM grids. Fixed samples were floated on drops of 2.5% w/v glutaraldehyde for 5 minutes. Grids were washed 10 times with distilled water, negative stained with 1% uranyl acetate for 1 minute, and air-dried. Grids were observed using a Hitachi 7700 transmission electron microscope operated at 80 kV. For NTA, exosome samples were diluted in PBS to achieve a particle concentration ranging from 10^7^/ml to 10^9^/ml and examined using a NanoSight NS300 (NanoSight Ltd., Amesbury, UK). The diluted samples of exosomes were injected into the laser chamber. The following settings were used for data acquisition: camera level 14, acquisition time 30 s, and detection threshold 4. Data analysis was performed with the NTA v3.2 software (NanoSight Ltd., Amesbury, UK).

### Characterization of exosomes

The ExoView Tetraspanin chip (ExoView, Boston, MA, USA) was arrayed with antibodies against proteins CD81, CD63, and CD9, markers of exosomes, and HLA IgG1 was used as a negative control. The sample was dropped onto the chip surface and placed at room temperature overnight. The cellular origin of the exosomes was analyzed using the ExoView Tetraspanin Labelling ABs (EV-TC-AB-01) of anti-CD81/ALEXA 555, anti-CD63/ALEXA 647, anti-CD9/ALEXA 488, anti-CD20 antigen-presenting cell (APC), and anti-CD56 APC. The images of isolated exosomes were analyzed using the ExoView R100 reader and the ExoScan 2.5.5 acquisition software (ExoView, Boston, MA, USA). The data were analyzed using ExoViewer 2.5.0 with sizing thresholds set to a diameter of 50 to 200 nm.

### Sequencing of exosomal mRNA

Total RNA was extracted from isolated exosome samples using the miRNeasy Serum/Plasma kit according to the manufacturer's instructions (Qiagen, Hilden, Germany). The quality and quantity of extracted RNA were evaluated using the Nanodrop 8000 UV-Vis spectrometer (NanoDrop Technologies Inc., Wilmington, DE, USA) and 4200 TapeStation Instrument (Agilent Technologies, Santa Clara, CA, USA). The RNA library was constructed using the TruSeq® RNA Access Library Prep Kit (Illumina Technologies., San Diego, CA, USA) according to the manufacturer's instructions. After pooled libraries were denatured, the sequencing of each library was performed by the 100-bp paired-end mode of the TruSeq Rapid PE Cluster Kit and TruSeq Rapid SBS Kit with HiSeq 2500 (Illumina Technologies., San Diego, CA, USA). The RNA-sequencing reads were aligned to the human reference genome (hg19) with STAR, and gene expression values were quantified using RNA-Seq by Expectation-Maximization (RSEM) 20. Subsequently, alignment statistics were generated using RNA-SeQC for quality control of these aligned reads. Gene fusions were predicted using several algorithms, including ChimeraScan, deFuse, FusionMap, MapSplice, and TopHat 21-25. For normalization of RNA sequencing data and comparison of exosomal mRNA expression among patients, the transcripts per million (TPM) value for each gene was generated as previously suggested 26. Using the results of exosomal mRNA sequencing, we analyzed the expression of genes previously reported as relevant with a subtype of NHL 27-29.

## Results

### Patients

A total of 33 patients with NHL comprised the study population of this study: diffuse large B-cell lymphoma (DLBCL, n = 17), intravascular B-cell lymphoma (IVL, n = 1), primary mediastinal large B-cell lymphoma (PMBL, n = 4), follicular lymphoma (FL, n = 3), mantle cell lymphoma (MCL, n = 3), and extranodal NK/T-cell lymphoma (ENKTL, n = 5). Among them, 25 patients were newly diagnosed, and 8 patients were enrolled after they relapsed (Supplement Table 1). With a median follow-up of 43.4 months (95% confidence interval: 37.0-49.8 months), 20 patients died due to disease relapse or progression, whereas the remaining 13 patients were alive at the time of analysis, including three relapsed patients (Supplement Figure 1B).

### Serum-derived exosomes

Exosomes were isolated from archived serum samples, and the concentrations of exosomes were not significantly different among them (Figure 1B). The size and morphology of the exosomes were not different, nor were the surface expressions of exosomal markers CD81, CD63, and CD9 (Figure 1C, D). After exosomes were sorted by the aforementioned exosomal markers, the surface expression of CD20, which is a marker for B-cells, showed enrichment of CD20-positive exosomes in the serum of patients with DLBCL, FL, and MCL, whereas exosomes derived from ENKTL patients showed CD56 expression, a marker for NK-cells (Figure 1F). The RNA was extracted from patients' serum-derived exosomes for exosomal mRNA sequencing, and the RNA concentration was not significantly different (Figure 1G). Before performing mRNA sequencing, we first conducted a whole transcriptomic sequencing of exosomal mRNA isolated from the DLBCL cell line U2932, which was obtained from Leibniz Institute DSMZ (German Collection of Microorganisms and Cell Cultures). The sequencing of U2932 exosomal mRNA identified the fusion of *RPS25/FOXR1* (Figure 1H). This result supported that exosomal cargo can contain the mRNA of tumor cells, and that exosomal mRNA sequencing could represent the mRNA expression of tumor cells because this fusion was confirmed in the U2932 cell line 30.

### Exosomal mRNA sequencing

**Figure 2:**
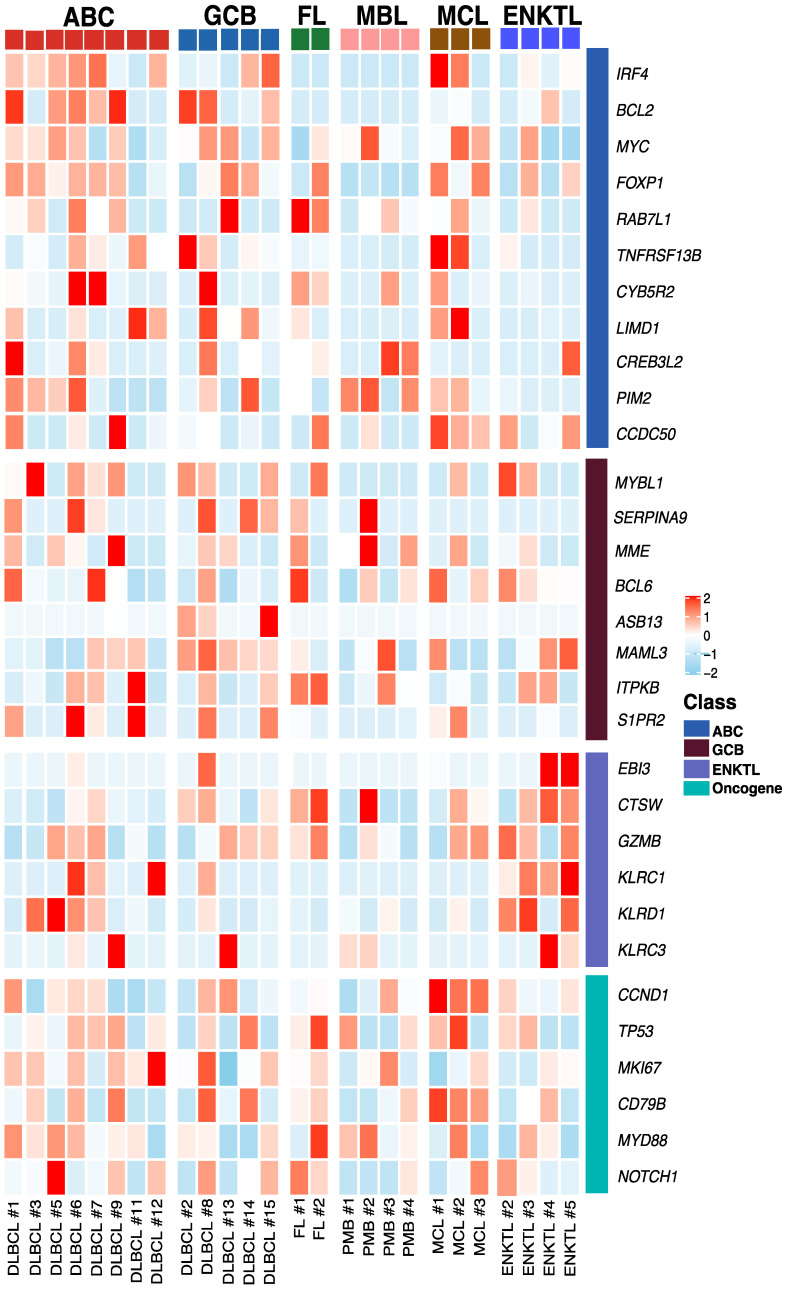
** Exosomal mRNA expression profile in patients with non-Hodgkin lymphoma** Heatmap of mRNA expression levels for selected genes at baseline. ABC (Activated B-cell like diffuse large B-cell lymphoma), GCB (Germinal center B-cell diffuse large B-cell lymphoma), FL (Follicular lymphoma), MBL (Primary Mediastinal B-cell lymphoma), MCL (Mantle cell lymphoma), ENKTL (Extranodal NK/T-cell lymphoma), DLBCL (Diffuse large B-cell lymphoma), PMB (Primary Mediastinal B-cell lymphoma)

Exosomal mRNA sequencing was performed successfully in 26 cases (79%, 26/33), whereas the remaining seven cases with less than 50 ng of RNA library failed to undergo RNA sequencing due to the amount of RNA library produced from exosomal RNA. Thus, exosomal mRNA sequencing was performed successfully in DLBCL (13/17), PMBL (4/4), MCL (3/3), FL (2/3), and ENKTL (4/5). The detection of exosomal mRNA was not significantly associated with stages, and the success rate of exosomal mRNA sequencing was not significantly different between newly diagnosed (76%, 19/25) and relapsed patients (88%, 7/8, P = 0.652) (Supplement Figure 1A). Among 25 patients with newly diagnosed NHL, the group with detected exosomal mRNA showed worse progression-free survival than the group without it (P = 0.027). The OS also showed similar pattern, although it failed to show statistical significance (P = 0.057, Supplement Figure 1C). Using exosomal mRNA sequencing data, we compared the expression genes that were essential for diagnosis and prognostication of DLBCL, PMBL, FL, MCL, and ENKTL, including *MYC, BCL2, BCL6, CCND1,* and genes belonging to the lymph2CX assay that are used commonly as a gene signature discriminating the cell of origin in DLBCL 27-29. The comparison of mRNA expression profiles showed different patterns consistent with activated B-cell-like (ABC) and germinal center (GC) type of DLBCL and PMBL, although the number of patients was small (Figure 2). The mRNA expression of *CCND1* was found in three MCL cases, consistent with cyclin D1 expression. The mRNA profiles of a relapsed FL patient (FL #2) showed higher expression of *TP53* and *MYD88* than those of a newly diagnosed FL patient (FL #1), and mRNA expression profiles of ENKTL were different from those of B-cell lymphomas, as expected (Figure 2).

### Post-treatment assessment of exosomal mRNA

**Figure 3:**
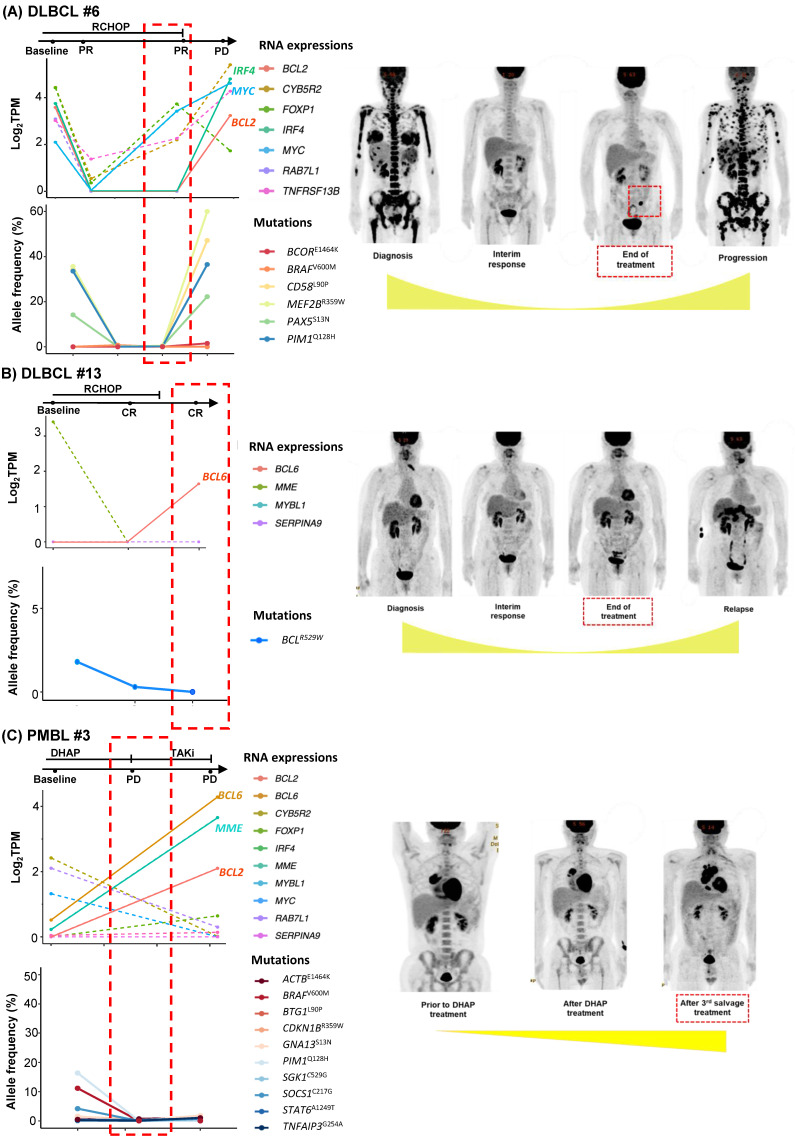
** Longitudinal changes in exosomal mRNA expression level in patients with diffuse large B-cell lymphoma (DLBCL) and primary mediastinal large B-cell lymphoma (PMBL). (A-C)** Exosomal mRNA expression was correlated more closely with clinical course than was that of ctDNA (red dashed box). The RNA expression level was evaluated by log_2_TPM. The ctDNA mutations were evaluated by allele frequency (%). Tumor volume is illustrated below the PET/CT images.

**Figure 4:**
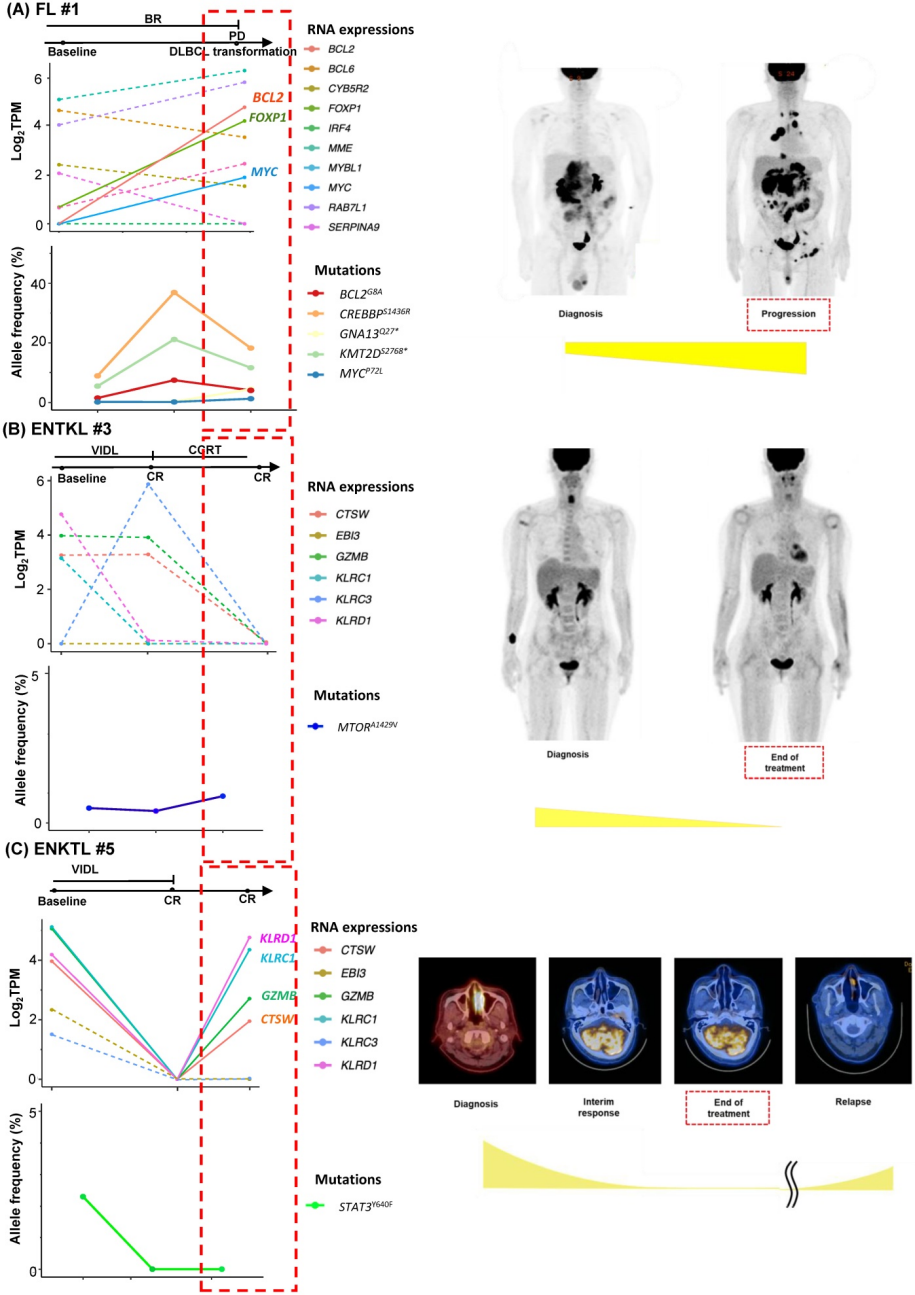
** Longitudinal changes in exosomal mRNA expression level in patients with follicular lymphoma (FL) and extranodal NK/T-cell lymphoma (ENKTL). (A)** The case of an FL patient whose histologic large cell transformation was confirmed. The expression of *BCL2* and* MYC* was newly detected in sEV mRNA profiles (red dashed box). **(B)** The case of an ENKTL patient who maintained a complete response without relapse. (C) The case of an ENKTL patient who showed late relapse after a complete response.

As we collected post-treatment samples as well as samples obtained at the time of relapse or progression, we performed longitudinal assessments of exosomal mRNA expression. When the serial changes of exosomal mRNA expression profiles were compared with their matched changes of ctDNA mutation profiles in cases who had both types of data available for comparison, the exosomal mRNA expression showed a closer association with disease relapse than did ctDNA mutations. Thus, in a case with ABC type DLBCL (DLBCL #6), the exosomal mRNA expression at diagnosis, such as *BCL2, IRF4,* and *MYC*, were persistent even after the completion of R-CHOP (rituximab, cyclophosphamide, doxorubicin, vincristine) chemotherapy, and it was consistent with the PET-CT findings showing a newly appeared lesion at the end of treatment. However, ctDNA mutations such as *BCOR, CD58, MEF2B, PAX5,* and *PIM1* were not detected during or after R-CHOP (Figure 3A). In a case with GC type DLBCL, the exosomal *BCL6* expression was newly observed at the end of treatment even though the PET-CT showed a complete response after completion of R-CHOP, whereas the *BCL6* mutation of ctDNA was decreased at the end of treatment. This patient eventually relapsed during follow-up (DLBCL #13, Figure 3B). When the exosomal mRNA was sequenced in a relapsed PMBL patient before and after the salvage treatment with DHAP (dexamethasone, cytarabine and cisplatin), the exosomal mRNA expression of *BCL2, BCL6,* and *MME* was increased at the time of disease progression after DHAP treatment, whereas the allele frequency of ctDNA mutations was not increased (PMBL #3, Figure 3C). This patient became refractory to the third salvage chemotherapy and eventually died. Exosomal mRNA sequencing of a newly diagnosed FL patient showed the expression of genes including *RAB7L1, CYB5R2, TNFRSF13B, MME,* and* BCL2* at the time of diagnosis. During bendamustine and rituximab treatment, the patient failed to respond. Based on the PET-CT findings, disease progression was confirmed, and a biopsy of the intra-abdominal lymph node showed large cell transformation. The blood sample was collected at the time of large cell transformation, and exosomal mRNA sequencing showed the increased expression of *BCL2, FOXP1,* and* MYC,* whereas no remarkable changes were observed in the ctDNA mutation profiles including *MYC* (Figure 4A). In a newly diagnosed patient with ENKTL, exosomal mRNA sequencing showed the loss of gene expression profiles that were initially detected at diagnosis after completion of planned treatment consisting of VIDL (etoposide, ifosfamide, dexamethasone, and L-asparaginase) followed by concurrent chemoradiotherapy (CCRT). This patient maintained a complete response even though the ctDNA showed *mTOR* mutation (Figure 4B). On the other hand, another patient with ENKTL showed an increase in exosomal mRNA expression including that of *CTSW, GZMB, KLRC1,* and* KLRD1,* even with a complete response based on PET/CT scan at the end of VIDL chemotherapy, although the ctDNA mutations showed complete resolution of the initially detected *STAT3* mutation (Figure 4C). This patient was monitored after completion of planned treatment; however, disease relapse eventually occurred.

## Discussion

In this study, we explored the feasibility of serum-derived exosomal mRNA sequencing in NHL patients and compared the clinical relevance of exosomal mRNA expression with that of ctDNA mutations as biomarkers for monitoring disease status such as detection of MRD. Although few studies exist regarding the feasibility of exosomal cargo mRNA as biomarkers for NHL patients, the importance of exosomes as a source of potential biomarkers, including tumor-derived RNAs, has been suggested because they can protect their cargo nucleic acids from degradation with the nuclease present in blood and other body fluids allowing transcriptome analysis of EV 31. Thus, analysis of exosomal mRNA actively released from living cells might have advantages compared to that of other biomaterials for liquid biopsy, especially ctDNA, which is released by necrotic or apoptotic cells 32. Actually, exosomal mRNA were reported to reflect the cell of origin in a previous study investigating the exosomes of DLBCL cell lines 33. Likewise, prior to conducting this study using patient serum samples, we identified the *RPS25/FOXR1* fusion in exosomal mRNA isolated from the DLBCL cell line U-2932, as previously reported 30.

Our study analyzing exosomes isolated from archived serum samples of NHL patients showed the performance of exosomal mRNA sequencing as a tool of exploration of mRNA extracted from exosomes, and exosomal mRNA expression profiling could be a feasible tool of liquid biopsy in NHL patients. However, exosomal mRNA sequencing was not successfully conducted in all patients, and it could be related to the relatively small amount of mRNA inside exosomes compared to the abundance of small RNAs such as microRNA 15, 16. Furthermore, exosomes isolated from blood could be mixed with normal cell-derived exosomes as well as tumor-derived exosomes 34. Indeed, identification of exosomal origins in our study showed that exosomes could originate from other cells circulating in blood because the fraction of CD20- or CD56-positive exosomes accounted for a relatively small portion of the total population of exosomes. Nevertheless, the exosomal mRNA expression profiles showed more enriched gene lists relevant to each subtype. These findings implied that the expression of specific genes could be substantial in exosomes even though the concentration generally is small in the blood 27, 28. In addition, considering that gene expression profiles were more informative than driver gene mutations in NHL, the role of exosome mRNA expression as a biomarker could be more relevant than that of ctDNA, which mainly targets gene mutations 35, 36. Accordingly, the serial changes of exosomal mRNA expression profiles were related more closely with disease status than ctDNA mutation profile (Figure 3, 4). Thus, the association of persistent exosomal mRNA expression at the end of treatment with disease relapse implied its potential as a biomarker for detection of MRD. Additionally, the information related with exosomal mRNAs expression could be useful for predicting the prognosis of patients because the previous study reported the poor prognosis of B-cell lymphoma patients with exosomal *BCL-6* and *C-MYC* mRNA at diagnosis and the association of exosomal *AKT* mRNA with the lack of response to rituximab-based treatment 37. Based on our results, the expression of genes that were increased frequently during disease progression or relapse, such as *BCL2, BCL6,* and* MYC* might be useful for monitoring disease status and predicting the risk of relapse.

The current study has several limitations. Most importantly, because of limited reports on exosomal mRNA in NHL patients, and lack of datasets, we could not validate our results. Additionally, relatively small number of patients may limit our data's interpretation and generalizability. Nevertheless, our data are clinically meaningful, as our results firstly offer the serial changes of serum derived exosomal mRNA expression during treatment course of NHL patients, comparing those of ctDNA mutations. Our results may be used to design future studies and guide clinical decisions. Future efforts will include more patients comparing the exosome test with some of the currently available blood-based assays.

Taken together, our results demonstrated the feasibility of exosomal mRNA expression profiles as a biomarker for NHL patients including DLBCL and compared its performance as a liquid biopsy with ctDNA mutation analysis using matched patients' samples. Although this study has limitations, including a relatively small number of patients, it provides rationale for continuation of studies to explore the potential of exosomal mRNA as a biomarker in NHL patients. Further study is warranted to validate and confirm our results.

## Supplementary Material

Supplementary figure and table.

## References

[1] Galimberti S, Genuardi E, Mazziotta F (2019). The Minimal Residual Disease in Non-Hodgkin's Lymphomas: From the Laboratory to the Clinical Practice. Front Oncol.

[2] Hu R, Winter A, Hill BT (2019). The Emerging Role of Minimal Residual Disease Testing in Diffuse Large B-Cell Lymphoma. Curr Oncol Rep.

[3] Cirillo M, Craig AFM, Borchmann S (2020). Liquid biopsy in lymphoma: Molecular methods and clinical applications. Cancer Treat Rev.

[4] Palmirotta R, Lovero D, Cafforio P (2018). Liquid biopsy of cancer: a multimodal diagnostic tool in clinical oncology. Ther Adv Med Oncol.

[5] Dogliotti I, Drandi D, Genuardi E (2018). New Molecular Technologies for Minimal Residual Disease Evaluation in B-Cell Lymphoid Malignancies. J Clin Med.

[6] Camus V, Jardin F (2019). Cell-free DNA and the monitoring of lymphoma treatment. Pharmacogenomics.

[7] Kwok M, Wu SP, Mo C (2016). Circulating Tumor DNA to Monitor Therapy for Aggressive B-Cell Lymphomas. Curr Treat Options Oncol.

[8] Jahr S, Hentze H, Englisch S (2001). DNA fragments in the blood plasma of cancer patients: quantitations and evidence for their origin from apoptotic and necrotic cells. Cancer Res.

[9] Kalluri R, LeBleu VS (2020). The biology, function, and biomedical applications of exosomes. Science.

[10] Raposo G, Stahl PD (2019). Extracellular vesicles: a new communication paradigm?. Nat Rev Mol Cell Biol.

[11] Thery C, Witwer KW, Aikawa E (2018). Minimal information for studies of extracellular vesicles 2018 (MISEV2018): a position statement of the International Society for Extracellular Vesicles and update of the MISEV2014 guidelines. J Extracell Vesicles.

[12] S ELA, Mager I, Breakefield XO (2013). Extracellular vesicles: biology and emerging therapeutic opportunities. Nat Rev Drug Discov.

[13] Yeh YY, Ozer HG, Lehman AM (2015). Characterization of CLL exosomes reveals a distinct microRNA signature and enhanced secretion by activation of BCR signaling. Blood.

[14] Azmi AS, Bao B, Sarkar FH (2013). Exosomes in cancer development, metastasis, and drug resistance: a comprehensive review. Cancer Metastasis Rev.

[15] Berardocco M, Radeghieri A, Busatto S (2017). RNA-seq reveals distinctive RNA profiles of small extracellular vesicles from different human liver cancer cell lines. Oncotarget.

[16] Lunavat TR, Cheng L, Kim DK (2015). Small RNA deep sequencing discriminates subsets of extracellular vesicles released by melanoma cells-Evidence of unique microRNA cargos. RNA Biol.

[17] Li M, Zeringer E, Barta T (2014). Analysis of the RNA content of the exosomes derived from blood serum and urine and its potential as biomarkers. Philos Trans R Soc Lond B Biol Sci.

[18] Zhu S, Ni Y, Sun G (2021). Exosomal TUBB3 mRNA expression of metastatic castration-resistant prostate cancer patients: Association with patient outcome under abiraterone. Cancer Med.

[19] Shin SH, Kim YJ, Lee D (2019). Analysis of circulating tumor DNA by targeted ultra-deep sequencing across various non-Hodgkin lymphoma subtypes. Leuk Lymphoma.

[20] Dobin A, Davis CA, Schlesinger F (2013). STAR: ultrafast universal RNA-seq aligner. Bioinformatics.

[21] Iyer MK, Chinnaiyan AM, Maher CA (2011). ChimeraScan: a tool for identifying chimeric transcription in sequencing data. Bioinformatics.

[22] McPherson A, Hormozdiari F, Zayed A (2011). deFuse: an algorithm for gene fusion discovery in tumor RNA-Seq data. PLoS Comput Biol.

[23] Ge H, Liu K, Juan T (2011). FusionMap: detecting fusion genes from next-generation sequencing data at base-pair resolution. Bioinformatics.

[24] Wang K, Singh D, Zeng Z (2010). MapSplice: accurate mapping of RNA-seq reads for splice junction discovery. Nucleic Acids Res.

[25] Kim D, Salzberg SL (2011). TopHat-Fusion: an algorithm for discovery of novel fusion transcripts. Genome Biol.

[26] Wagner GP, Kin K, Lynch VJ (2012). Measurement of mRNA abundance using RNA-seq data: RPKM measure is inconsistent among samples. Theory Biosci.

[27] Scott DW, Wright GW, Williams PM (2014). Determining cell-of-origin subtypes of diffuse large B-cell lymphoma using gene expression in formalin-fixed paraffin-embedded tissue. Blood.

[28] Hans CP, Weisenburger DD, Greiner TC (2004). Confirmation of the molecular classification of diffuse large B-cell lymphoma by immunohistochemistry using a tissue microarray. Blood.

[29] Huang Y, de Reyniès A, de Leval L (2010). Gene expression profiling identifies emerging oncogenic pathways operating in extranodal NK/T-cell lymphoma, nasal type. Blood.

[30] Pommerenke C, Hauer V, Zaborski M (2016). Chromosome 11q23 aberrations activating FOXR1 in B-cell lymphoma. Blood Cancer J.

[31] Turchinovich A, Drapkina O, Tonevitsky A (2019). Transcriptome of Extracellular Vesicles: State-of-the-Art. Front Immunol.

[32] Krug AK, Enderle D, Karlovich C (2018). Improved EGFR mutation detection using combined exosomal RNA and circulating tumor DNA in NSCLC patient plasma. Ann Oncol.

[33] Rutherford SC, Fachel AA, Li S (2018). Extracellular vesicles in DLBCL provide abundant clues to aberrant transcriptional programming and genomic alterations. Blood.

[34] Whiteside TL (2018). The emerging role of plasma exosomes in diagnosis, prognosis and therapies of patients with cancer. Contemp Oncol (Pozn).

[35] Heitzer E, Haque IS, Roberts CES (2019). Current and future perspectives of liquid biopsies in genomics-driven oncology. Nat Rev Genet.

[36] Reddy A, Zhang J, Davis NS (2017). Genetic and Functional Drivers of Diffuse Large B Cell Lymphoma. Cell.

[37] Provencio M, Rodríguez M, Cantos B (2017). mRNA in exosomas as a liquid biopsy in non-Hodgkin Lymphoma: a multicentric study by the Spanish Lymphoma Oncology Group. Oncotarget.

