# Supplementary material for: Clinical relevance of serum-derived exosomal messenger RNA sequencing in patients with non-Hodgkin lymphoma
Source: J Cancer. 2022 Feb 21;13(5):1388–97. doi: 10.7150/jca.69639 (PMC8965112; doi:10.7150/jca.69639)

Supplement Table 1. Clinical characteristics of the study patients

Variables	Total cohort (n = 33)
Age (years) (median)	56 (25-83)
Age ≥ 60 years	13 (39.4%)
Sex: male	18 (54.5%)
Types of cancer	
DLBCL*	17 (51.5%)
IVL*	1 (3.0%)
PMBL*	4 (12.1%)
FL *	3 (9.1%)
MCL*	3 (9.1%)
ENKTL*	5 (15.2%)
Ann Arbor Stage	
II	5 (15.2%)
III	12 (36.4%)
IV	16 (48.5%)
Disease status	
Newly diagnosed disease	25 (75.8%)
Relapsed disease	8 (24.2%)

Data are presented as no. (%) or median (range).

* DBLCL: Diffuse large B cell lymphoma; IVL: intravascular B-cell lymphoma; PMBL: primary mediastinal large B-cell lymphoma; FL: follicular lymphoma; MCL: mantle cell lymphoma; ENTKL: extranodal NK/T-cell lymphoma.

Supplementary figure 1

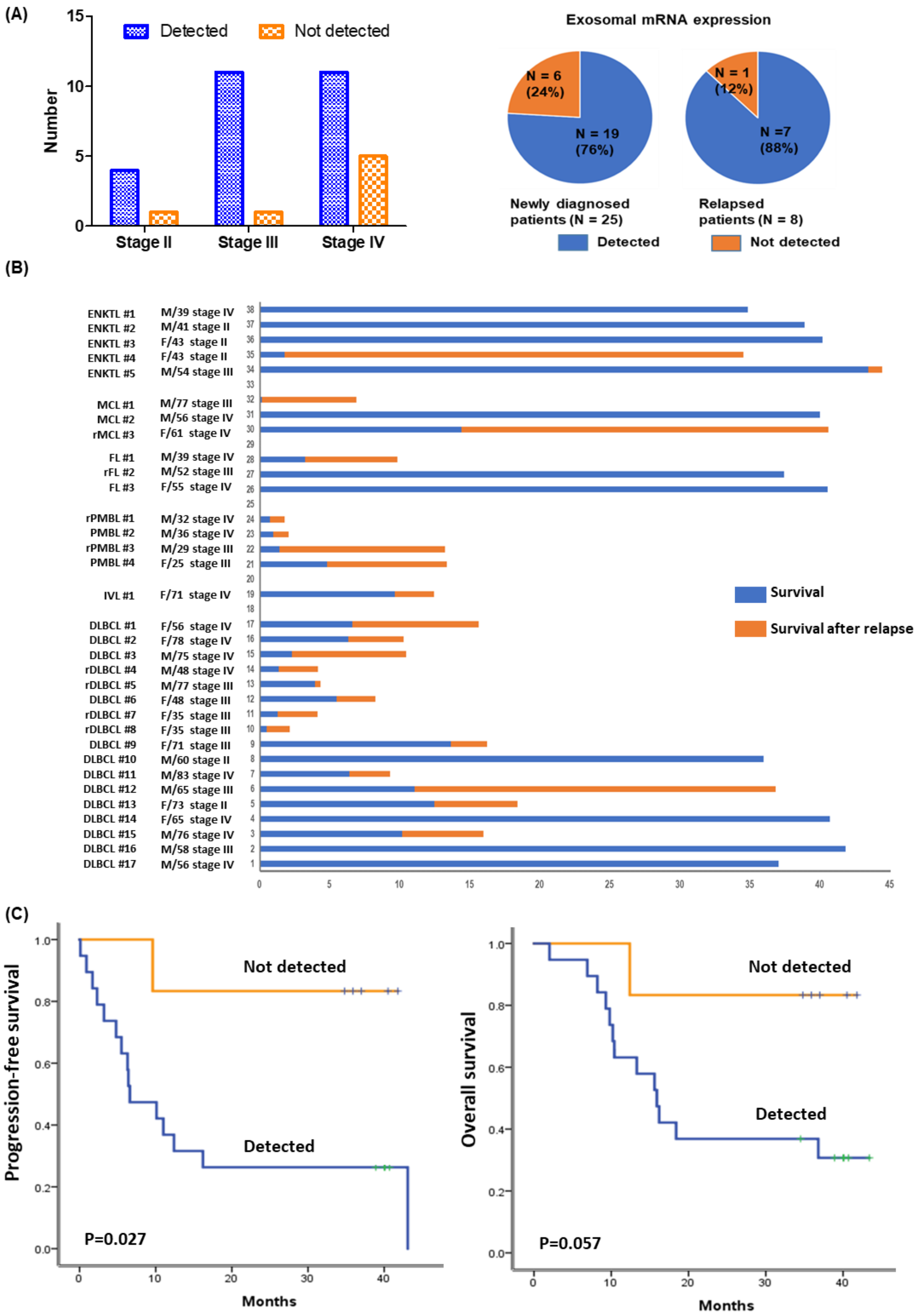

Supplement: Supplementary file 1 — Supplementary figure and table. [file jcav13p1388s1.pdf]
